# Plasmacytoid bladder cancer: a rare case report

**DOI:** 10.1097/MS9.0000000000000374

**Published:** 2023-04-11

**Authors:** Wajdi Benabdallah, Mouna Ben Othmane, Ines Ouahchi, Sarra Mestiri, Oussama Belkacem, Khaireddine Bouassida, Wissem Hmida, Mehdi Jaidane

**Affiliations:** a Urology Department; b Pathology Department, Sahloul Hospital; c Department of Cytogenetic and Reproductive Biology, Farhat Hached University Teaching Hospital, Sousse, Tunisia

**Keywords:** bladder cancer, case report, plasmacytoid variant, urothelial carcinoma

## Abstract

Plasmacytoid carcinoma of the bladder is a rare histological variant of urothelial carcinoma associated with late presentation. This pattern of disease may portend an extremely poor prognosis and significant challenges for treatment with curative intent.

**Case presentation:**

The authors report a case of a patient with locally advanced plasmacytoid urothelial carcinoma (PUC) of the bladder. A 71-year-old man with a history of chronic obstructive pulmonary disease presented with gross hematuria. The rectal examination revealed a fixed bladder base. A computed tomography scan showed a pedunculated lesion arising from the anterior and left lateral bladder wall and extended to the perivesical fat. The patient underwent transurethral resection of the tumor. The histologic examination revealed the presence of muscle-invasive PUC of the bladder. The decision of the multidisciplinary consultation meeting was to do palliative chemotherapy. Thus, the patient could not receive systemic chemotherapy and died 6 weeks after transurethral resection of the bladder tumor.

**Clinical discussion:**

A plasmacytoid variant of urothelial carcinoma is a rare subtype of urothelial carcinoma that has a poor prognosis with a high mortality rate. The disease is usually diagnosed at an advanced stage. Given the rarity of plasmacytoid bladder cancer, treatment guidelines are not clear; therefore, more aggressive treatment may be required.

**Conclusion:**

PUC of the bladder is characterized by high aggressiveness, an advanced stage at the time of diagnosis, and a poor prognosis.

## Introduction

Highlights

- Plasmacytoid bladder cancer is a rare carcinoma with a poor prognosis.
- The therapeutic approach for this pathology remains in discussion due to its rarity.
- The challenges of curative treatment with rapidly progressive cancer.

Plasmacytoid urothelial carcinoma (PUC) is an extremely rare and aggressive variant of urothelial carcinoma. Its first description was made by Sahin *et al.* in 1991, and it was recognized by WHO in 2004. The prognosis of this variant is often poor due to the commonly advanced disease presence upon diagnosis, warranting aggressive treatments^1^. This case report has been reported in line with the SCARE (Surgical CAse REport) Criteria^2^.

## Case report

A 71-year-old man with severe chronic obstructive pulmonary disease, poor adherence to treatment, and active smoking presented with gross hematuria and generalized fatigue.

Physical examination revealed normal vital signs; a soft, nontender abdomen and no palpable abdominal mass. The rectal examination revealed a fixed bladder base.

A laboratory examination was performed: the hemoglobin was 6.6 g/dl (13.8–17.2), leukocyte count was 14 600 (5000–10 000) with normal renal function, and creatinine was 74 µmol/l (65.4–119.3 μmol/l).

**Figure 1:**
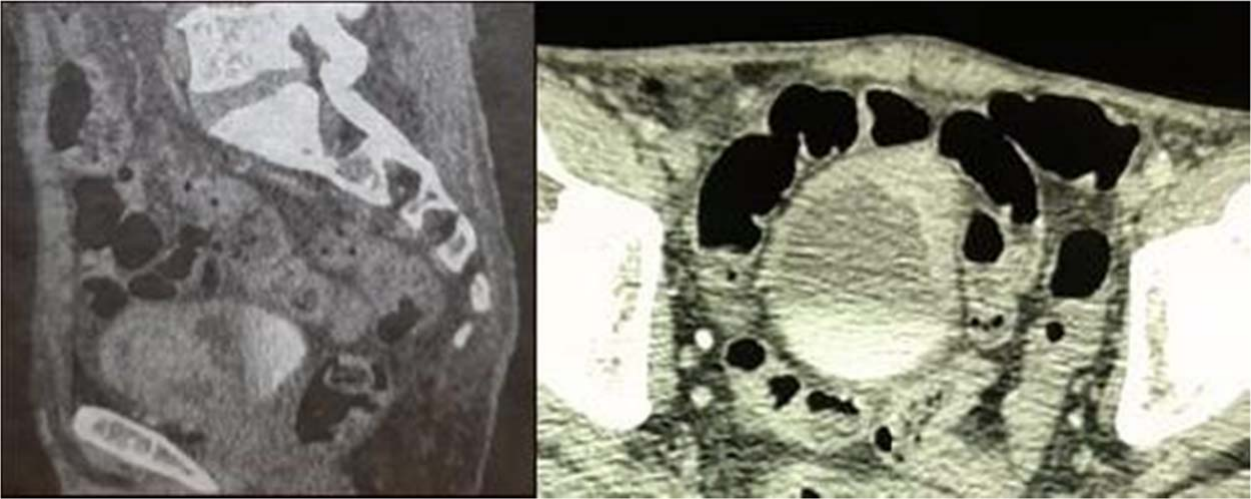
Computed tomography scan showing an enhancing, pedunculated lesion arising from the anterior and left lateral bladder wall (plasmacytoid urothelial carcinoma of the bladder).

A computed tomography chest, abdomen, and pelvic scan (Fig. 1) demonstrated an enhancing, pedunculated lesion arising from the anterior and left lateral bladder wall measuring 18×22×20 mm and extended to the perivesical fat, left internal iliac lymph node, the presence of panlobular emphysema and pulmonary fibrosis without distant metastasis.

**Figure 2:**
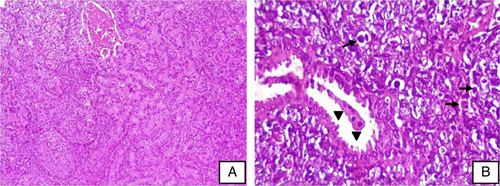
Photomicrographs of bladder high-grade urothelial carcinoma showing (A) glandular differentiation (hematoxylin and eosin, H&E ×40); (B) discohesive tumor cells with scant and eccentric cytoplasm mimicking plasma cells (arrow). Note the presence of prominent apical snouts (arrowhead) (H&E ×400).

The preanesthetic consultation was done. The patient was prepared for surgery and transfused a total of two units of packed red blood cells, then underwent cystoscopy and complete transurethral resection of a large, solid, and infiltrating tumor arising from the anterior and left lateral bladder wall. This operation was performed in our department of urology, a university hospital, by a professor of urology. Multiple resected fragments of bladder tumor were addressed to the pathology department. On histopathological examination (Fig. 2), the fragments were diffusely invaded by a high-grade malignant neoplasm extending from the mucosa to the muscularis propria. The tumor consisted of large nests and cords, with the focal glandular formation and discohesive neoplastic cell sheet with plasmacytoid features. These cells are a high nuclear cytoplasmic ratio with eosinophilic cytoplasm and eccentric enlarged nuclei. The glands were lined by a single layer of neoplastic columnar cells arranged around a lumen with prominent apical snouts and mucin production. Numerous lymph and blood vessel tumor emboli were observed.

The diagnosis of a locally advanced PUC of the bladder was made. The patient was discharged on the third postoperative day under analgesic treatment and a telephone follow-up was organized. The decision of the multidisciplinary consultation meeting was to do palliative chemotherapy. The patient was contacted by telephone to organize his oncological treatment. Thus, he could not receive systemic chemotherapy and died 6 weeks after transurethral resection of the bladder tumor.

## Discussion

The plasmacytoid variant of urothelial carcinoma is a rare subtype of urothelial carcinoma that has a poor prognosis. The mean age of initial diagnosis is 63 years (range 45–78), and the constituent ratio shows a male predominance^3^. The most common presenting symptom for diagnosis is hematuria. In our case, the patient is 71 years old and presented with gross hematuria. Pelvic peritoneal infiltration by PUC can be detected in radiology as thick sheets extending along the fascial planes and is suggested to be a characteristic imaging finding^4^. Cystoscopic findings are usually mucosal induration, thickened vesical wall, and focal masses. Therefore, the diagnosis of the PUC is purely histological, with the presence of small cells with the hyperchromatic eccentric nucleus and whose abundant eosinophilic cytoplasm contains very small mucous vacuoles resembling a plasma cell. The therapeutic approach for this pathology remains in discussion due to its rarity. The disease is usually diagnosed at an advanced stage. Dayyani *et al*.^3^ report a median overall survival of 45.8 months in 14 patients who received neoadjuvant or adjuvant chemotherapy for cystectomy. In this last series, the authors did not note any significant difference between the survival time of patients who received neoadjuvant chemotherapy and patients initially treated with cystectomy. Diamantopoulos *et al*.^5^ reports a poor pathologic response to neoadjuvant chemotherapy and inferior outcomes when compared to conventional urothelial. For the locally advanced PUC, a radical cystectomy is considered the primary treatment. Veskimäe *et al*.’s^6^ systematic review indicated neoadjuvant chemotherapy for the PUC appeared to be beneficial, so neoadjuvant cisplatin-based chemotherapy may be offered. Most authors propose cisplatin-based chemotherapy for patients presented initially with the metastatic or locally unresectable disease with an overall response rate exceeding 50%^3^,^7^. In our case, the patient could not receive systemic chemotherapy and died 6 weeks after transurethral resection of the bladder tumor. It is difficult to define the optimal treatment strategy for PUC of the bladder because the disease is usually diagnosed at an advanced stage. Patients with plasmacytoid variant-urothelial carcinoma of the bladder had a higher frequency of stage pT3 or greater [odds ratio (OR) 3.84, 95% CI: 1.63–9.03; *P*=0.002] and risk of lymph node metastasis (OR 2.58, 95% CI: 1.15–5.76; *P*=0.02), ureteral margin positive (OR 12.18, 95% CI: 4.62–32.13; *P*<0.00001) and perivesical soft tissue margin positive (OR 12.31, 95% CI: 5.15–29.41, *P*<0.00001) status after radical cystectomy than those with conventional urothelial carcinoma of the bladder^1^. Finally, early diagnosis is challenging and arguably the most important prognostic factor in influencing survival.

## Conclusion

The PUC of the bladder is a rare, high-grade variant of urothelial carcinoma. The prognosis remains poor, with few long-term survivors. However, the optimal treatment for patients with PUC of the bladder remains unsettled due to the rarity of evidence. Further well-designed studies are needed to clarify the importance of a comprehensive treatment strategy in patients with this aggressive variant of urothelial carcinoma.

## Ethical approval

Ethical approval is waived at our institution.

## Consent

Written informed consent was obtained from the family of the patient for the publication of this case report and accompanying images. A copy of the written consent is available for review by the Editor-in-Chief of this journal on request.

## Sources of funding

This research did not receive any specific grant from funding agencies in the public, commercial, or not-for-profit sectors.

## Author contribution

W.B.: writing – original draft; M.B.O., S.M., and O.B.: writing and editing; I.O. and K.B.: review and editing; W.H.: supervision and reviewing; M.J.: supervision and review.

## Conflicts of interest disclosure

The authors declare that they have no conflicts of interest.

## Guarantor

Wajdi Benabdallah.

## Provenance and peer review

Not commissioned, externally peer-reviewed.
